# Dental Prosthesis

**Published:** 1883-07

**Authors:** Fr. Kleinmans

**Affiliations:** Fleusburg


### DENTAL PROSTHESIS.

BY FR. KLEINMANN, OF FLEUSBURG.

In replacing an artificial nose the four following points are to be observed:

1. The impression of the face;
2. Modeling of the nose;
3. The making of the same;
4. The attachment.

### I—THE IMPRESSION.

The larger the deficiency, and the more movable the parts are, the more difficult it is to obtain a perfect impression. Dr. Bruck, who has replaced four artificial noses, suggests the following: "I first take an impression of the defective parts in soft wax, which I afterward place in cold water to harden. Then I pour thin plaster into this wax shape, and softly squeeze it upon the deficiency, holding it in place until the plaster is perfectly hard. I then remove it, and into this mask I again pour plaster, thereby obtaining the natural model of the deformity. In order to avoid the adhering of the impression material to the skin, I give the corresponding parts a thin coat of oil or vaseline, and if a mustache is present I cover it with oiled paper.

II—MODELING OF THE NOSE.

Dr. Bruck justly remarks that the greatest efforts of the artist ought to be directed to shaping of the nose, which must be in accurate correspondence with the face of the patient. There are various ways to accomplish this. Dr. Grohnwald had the model of the artificial substitute made of clay by Professor Sumering, the sculptor. Dr. Bruck procured a man whose physiognomy was similar to that of the patient, and shaped the nose in accordance with that nose. I procured a mask, oiled the inner side, and filled it with plaster. After the plaster had perfectly set I placed it in water, when the mask became detached from the thus obtained perfect model of a face. The nasal portion of this moist plaster model I coated with wax, which after it had hardened could be removed.

III—THE MAKING OF THE NOSE.

Various substances have been made use of, such as wax, pasteboard, leather, parchment, wood and silver, but at the present time rubber and celluloid have the preference. Dr. Lefoulon, of Paris, about twenty years ago, recommended the casting of a dye and counter dye, upon which a gold or platinum plate might be stamped. The outer surface of this metallic nose was to be enameled to correspond with the color of the face. When rubber is employed the question arises, shall we use hard or soft rubber? Both have advantages. Hartung made a nose of hard pink rubber. Bruck finds soft rubber to be the best material. Zimmer made the nose of pink rubber and had it painted. Grohnwald made an artificial substitute of celluloid. I have tried both hard and soft rubber, but have decided in favor of the celluloid. After I have fitted the wax nose with its gold spring upon the patient's face, I place the same with its tip downward in a vessel containing cotton, and then fill the inner part of this wax nose with thin plaster. After the plaster has become hard I invest the whole in a flask, putting the splint downward, and the nose tip upward, then filling up the rest of the flask in the usual manner. After the wax has been washed off I place a previously pressed celluloid nose in its place, and close the flask. In order to give the outer surface of the nose a rough appearance, a piece of linen is laid between the plaster and outer surface of the celluloid nose. I do not polish the nose, as the paint will adhere and keep much better without it. The making of a nose of soft rubber is, according to Dr. Bruck's system, a little more complicated. Upon the model he presses unvulcanized rubber, imbeds and vulcanizes it. This so formed hard rubber nose is to be accurately finished and fitted, in accordance with the patient's physiognomy. To obtain the soft rubber nose imbed the hard rubber one in plaster in the same manner as above described, but use a little alum that the plaster may set very hard. Grohnwald vulcanizes soft rubber for thirty minutes. The coloring of the nose he leaves to an artist.

### IV—THE ATTACHMENT.

The most natural and at the same time the best attachment, is by means of an obturator, as in the cases related by Hartung and Grohnwald. In most instances, however, we have to depend for the support upon a pair of spectacles. Zimmer, besides the hold obtained by the spectacles, applies a pair of hard rubber strips. My method, which I consider new, is as follows: a splint or spring, about two inches in length, closely fitting the lateral walls of the anterior nares, and made of hard gold wire about one millimeter in thickness, is fastened on the inner side of the nose tip. The posterior extremities of the splint are bent into eyelets, which, when in position do not quite reach the posterior nares. The thin margin of the celluloid substitute held in position by this spring, soon imbeds itself into the tissues upon which it rests. The nose, together with its surrounding skin, is then coated with collodion, which imparts to it a very life-like appearance. The attachment by collodion alone is not safe, as Dr. Grohnwald had a patient who lost a nose by sneezing, which had been attached in the above manner.

